# A Unified Fourth-Order Tensor-Based Smart Community System

**DOI:** 10.3390/s20215990

**Published:** 2020-10-22

**Authors:** Chang Liu, Huaiyu Wu, Junyuan Wang, Mingkai Wang

**Affiliations:** 1 College of Design and Innovation, Tongji University, 281 Fuxin Road, Shanghai 200092, China; liuc@tongji.edu.cn; 2 Department of Information and Communication Engineering, College of Electronics and Information Engineering, Tongji University, 4800 Cao’an Highway, Shanghai 201804, China; why_not@tongji.edu.cn; 3 Institute for Advanced Study, Tongji University, 1239 Siping Road, Shanghai 200092, China; 4 School of Mathematical Sciences, Tongji University, 1239 Siping Road, Shanghai 200092, China; wmk@tongji.edu.cn

**Keywords:** tensor modeling, smart community system, Internet of Things

## Abstract

Empowered by the ubiquitous sensing capabilities of Internet of Things (IoT) technologies, smart communities could benefit our daily life in many aspects. Various smart community studies and practices have been conducted, especially in China thanks to the government’s support. However, most intelligent systems are designed and built individually by different manufacturers in diverging platforms with different functionalities. Therefore, multiple individual systems must be deployed in a smart community to have a set of functions, which could lead to hardware waste, high energy consumption and high deployment cost. More importantly, current smart community systems mainly focus on the technologies involved, while the effects of human activity are neglected. In this paper, a fourth-order tensor model representing object, time, location and human activity is proposed for human-centered smart communities, based on which a unified smart community system is designed. Thanks to the powerful data management abilities of a high-order tensor, multiple functions can be integrated into our system. In addition, since the tensor model embeds human activity information, complex functions could be implemented by exploring the effects of human activity. Two exemplary applications are presented to demonstrate the flexibility of the proposed unified fourth-order tensor-based smart community system.

## 1. Introduction

Community, as a social unit with commonality, has been increasingly complex and significant due to the issues and concerns brought by social problems and environmental changes. In China, the term of community (*shequ* in Chinese) is used in an official governmental discourse, which is designated as the basic unit of urban social, political and administrative organization within a certain geographic area and a certain population [1,2]. With the rapid increase of the urban population in China, the concentration of urban functions and resources in the city center has posed plenty of challenges in transportation, medical care, education, housing, etc. This year, the COVID-19 pandemic outbreak brought Chinese bustling cities to an unexpected halt. In order to ease pressure at hospitals and other public service institutes, communities started playing an important role in city governance and management. A typical example is to monitor the body temperatures of the residents and report to the government immediately if a fever is detected, which requires extensive repetitive work. This urges the development of a human-centered smart community system that could continuously monitor and quickly react to abnormal events by taking advantage of Internet of Things (IoT) technologies [3,4].

In China, seven cities/districts that engage different partners and the public in the determination process have been designated as smart communities [5] to implement intelligent applications in city management, public services, medical care and industrial development [6]. By connecting various smart communities together via wireless/wired broadband networks, remote access to many services across the whole country or even worldwide would be possible, e.g., consultation of medical care and education. At the same time, satellite cities and new communities have been built to enhance the smart community pilot program so as to increase efficiency, improve connectedness and centralize information on residents from public finance, taxation, city planning, housing, commerce, education and justice. For example, [7] applied the PROMETHEE-II algorithm in Industrial 4.0 to help with the selection of Intelligent Internet of Things (IIoT) platforms. A distributed demand side management system was proposed in [8] to facilitate smart energy trading in a local community. [9] discussed the vision of a holistic smart city that takes advantage of crowdsourced data to make customizable and scalable decisions. 

A common fact underlies the practices: that is, these communities/cities are meeting a growing demand for being interconnected and intelligent and are labeled as “smart”. However, in smart communities’ research and practices, we see the gap between visions and facts. Existing smart communities are mainly practiced in a new town/city, where few people live before being built. As a result, the core of these smart communities is the intelligent infrastructure, including deploying sensors and connecting them to the Internet. A majority of studies discussed individual diverging smart systems based on different IoT platforms and cloud computing technologies, e.g., medical care, e-governance, job creation, etc. [10,11,12,13,14]. In order to meet the diverse requirements in smart communities, a complex intelligent system that consists of multiple individual subsystems operating independently will need to be deployed, which could lead to significant wasting of digital technological equipment. How to build a unified smart community system to enable flexible add-on functions still remains largely unknown.

On the other hand, a number of scholars raised questions about whether the overuse and overemphasis of digital technologies in shaping communities and cities is beneficial for enabling a livable environment [15,16]. One of the problems is that the current smart communities are designed with the focus on the optimization of technologies, while people’s needs from different stakeholders have been rarely taken into account. 

**Figure 1:**
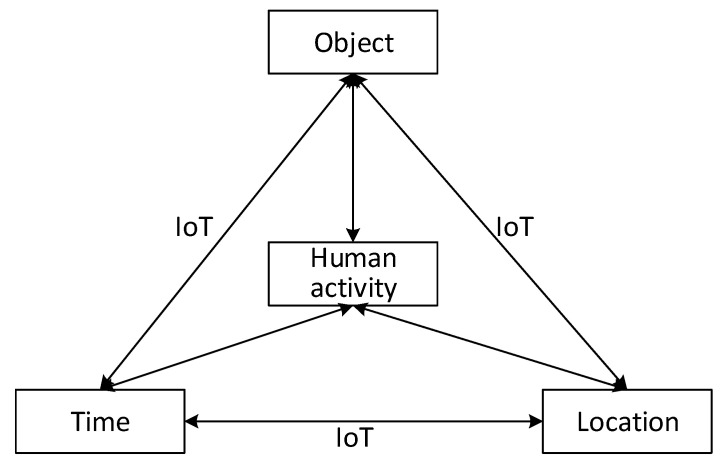
Four-dimensional model of smart community system.

In this paper, we consider a human-centered smart community, which serves humans’ needs based on systematic information. For this purpose, a four-dimensional model is built, as depicted in Figure 1, which is based on object, time, location and human activity. The relationship between any two of them can be reflected by the data collected in the IoT network. For these four dimensions, *location* means a particular place or position in the community. *Object* refers to things and people that exist independently. *Time* is a series of consecutive discrete timestamps. Furthermore, *human activities* are various actions taken by people for commuting, living, reaction or necessity, such as running, walking, standing, talking, sitting, falling, fighting, abnormal activities, etc. With the help of IoT, data can be collected from wearable sensors, or remotely recorded from video, radar and other wireless sensing methods. 

Based on the above four-dimensional model of a human-centered smart community system, a fourth-order tensor model that represents object, time, location and human activity is proposed in this paper to store all the data collected by IoT devices. This is motivated by the appealing features of the tensor to manage heterogeneous and enormous data [17,18], which has also been shown to be a powerful tool for public bicycle rental forecasting [19] and breathing monitoring [20], to name a few. As all the data are now formalized into the proposed fourth-order tensor model, a unified smart community system can be proposed, where tensor data can be truncated and extracted to fit various application purposes. These retrieved data could be then processed in parallel. It can be seen that such a fourth-order tensor-based smart community system provides a systematic solution to various living needs in smart communities, and thus avoids the use of multiple application-driven intelligent systems. Therefore, a couple of benefits can be obtained: (1) resources could be saved as a number of key devices can be shared by multiple application functions, e.g., gateways, which also reduces energy consumption; (2) functions can be easily removed or added, enabling a flexible and extendable smart community system, which is especially crucial for old towns/cities where deployment of new equipment is usually complicated and of high cost. In addition, compared to the traditional schemes based on third-order tensors, extra information could be obtained and thus gains could be achieved by adopting our proposed fourth-order tensor-based smart community system. 

The rest of this paper is organized as follows. Section 2 models the collected data in smart communities as a fourth-order tensor and proposes a unified tensor-based smart community system. Two application examples are then demonstrated in Section 3. Section 4 discusses the key findings, and concluding remarks are summarized in Section 5. 

## 2. Methods

In this section, all the data collected from intelligent sensors are formalized into a fourth-order tensor, based on which a unified smart community system is proposed. Before presenting the fourth-order-based smart community system, let us first introduce some useful definitions regarding tensor [21].

### 2.1. Basic Definitions

**Definition** **1 (Tensor).** *A tensor is a multidimensional array. The order of a tensor is its number of dimensions, while the dimensions are usually referred to as modes or ways. An Nth-order tensor is denoted as* $𝓧\in {\mathbb{R}}^{I_{1}\times I_{2}\times \cdots \times I_{N}}$ *, where* $I_{n}$ *is the size of mode n. Particularly, a zeroth-order tensor is a scalar, a first-order tensor is a vector, and a second-order tensor is a matrix.*

**Figure 2:**
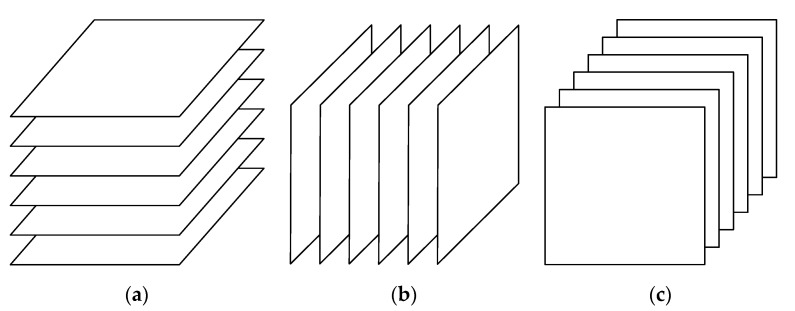
Graphic illustration of slices of a third-order tensor 𝓧. (**a**) Horizontal slices ${𝓧}_{i_{1}\;:\;\;:}$; (**b**) Lateral slices ${𝓧}_{\;:\;i_{2}\;:}$; (**c**) Frontal slices ${𝓧}_{\;:\;\;:\;i_{3}}$.

**Definition** **2 (Slice).** *A two-dimensional section of a tensor, whose all but two indices are fixed, is referred to as a slice. For example, a third-order tensor* $𝓧\in {\mathbb{R}}^{I_{1}\times I_{2}\times I_{3}}$ *has the horizontal (mode 1) slices* ${𝓧}_{i_{1}\;:\;\;:}$ *, the lateral (mode 2) slices* ${𝓧}_{\;:\;i_{2}\;:}$ *, and the frontal (mode 3) slices* ${𝓧}_{\;:\;\;:\;i_{3}}$ *, as illustrated in Figure 2.*

**Definition** **3 (Mode-*n* Product).** *A mode-n product or n-mode product of tensor* $𝓧\in {\mathbb{R}}^{I_{1}\times I_{2}\times \cdots \times I_{N}}$ *and a matrix* $U\in {\mathbb{R}}^{I_{n}\times J}$ *is denoted by* $𝓨=𝓧\times_{n}U$ *with* $𝓨\in {\mathbb{R}}^{I_{1}\times \cdots \times I_{n-1}\times J\times I_{n+1}\times \cdots \times I_{N}}$ *, and*

(1) $${𝓨}_{i_{1}\cdots i_{n-1}ji_{n+1}\cdots i_{N}}=\sum_{i_{n}=1}^{I_{n}}x_{i_{1}i_{2}\cdots i_{N}}\cdot u_{ji_{n}}.$$

*It is obvious that with the mode-n product, each mode-n fiber (which is defined by fixing every index but one) of the tensor is multiplied by the matrix **U**.*

### 2.2. Fourth-Order Tensor Modeling of Smart Community Data

In this section, a fourth-order tensor will be constructed to encode all the data in the four dimensions of object, time, location and human activity. As the object, time and location information can be usually obtained directly from intelligent sensors, let us first construct a third-order tensor to represent them and the interplay among them, after which the fourth dimension, human activity, will be added.

**Figure 3:**
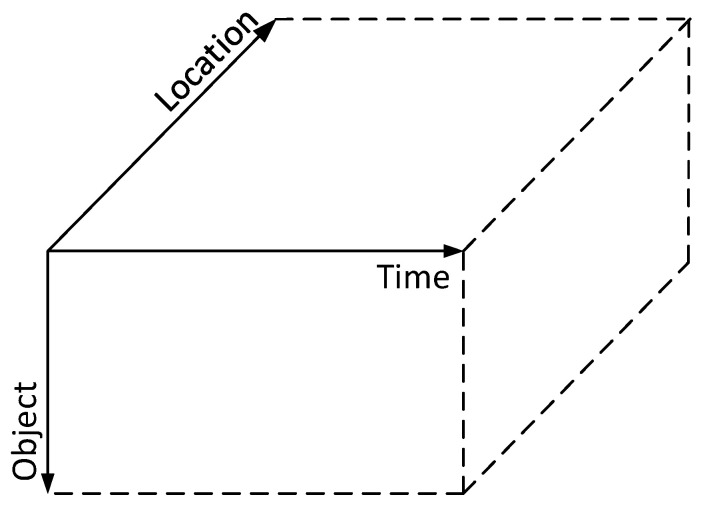
Graphic illustration of the third-order tensor with 3 modes representing object, location and time, respectively.

Let $𝓧\in {\mathbb{R}}^{I\times J\times K}$ denote the third-order tensor of object, time and location, as illustrated in Figure 3. Specifically, the indices of the object dimension (mode 1) are the object indices numbered according to the predefined policy. For example, the objects located at fixed positions, such as surveillance cameras and environmental sensors, are assigned fixed unique indices, while a moving object is assigned a dynamic index once successfully recognized from surveillance video images. This is because visitors and new objects could appear from time to time in smart communities, making it impossible to assign a fixed unique index for every object. 

The second mode is the time dimension with indices being timestamps, whose resolution depends on the sampling frequency of sensors and the requirement of specific applications. For a lower temporal resolution requirement, the time-mode data could be sampled again at the preprocessing stage to reduce the amount of data so as to speed up further processing. 

**Figure 4:**
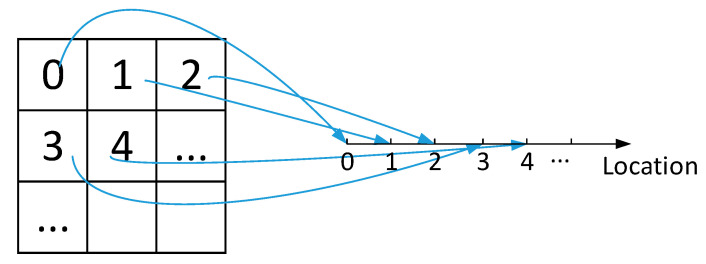
A simple location coding scheme.

Finally, the indices of location dimension (mode 3) are the codes representing different geographic points/areas by applying a carefully designed coding scheme to transform the three-dimensional space into discrete sequences. A detailed discussion around the location coding scheme design could be found in [22] and the references therein, which is out of the scope of this paper. For the sake of demonstration, a simple location coding scheme is shown in Figure 4, where the whole monitored area is divided into *K* square subareas of equal size and numbered from the left-hand side to the right-hand side first and then from up to down. 

**Figure 5:**
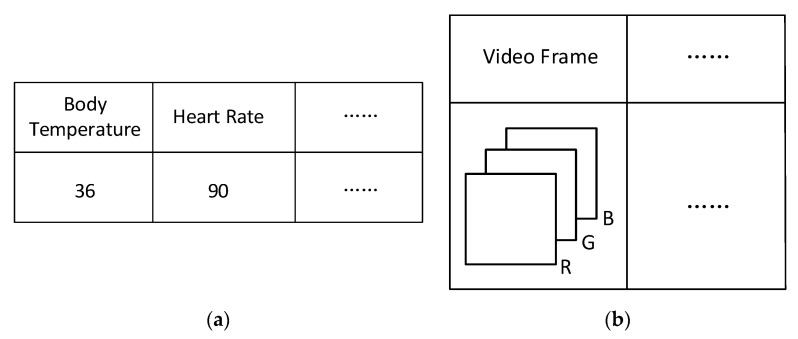
Illustrative toy examples of an element of a third-order tensor. (**a**) ${𝓧}_{ijk}$ for human object *i* at timestamp *j* and location *k*; (**b**) ${𝓧}_{i^{\prime }j^{\prime }k^{\prime }}$ for surveillance camera object $i^{\prime }$ at timestamp $j^{\prime }$ and location $k^{\prime }$.

An element ${𝓧}_{ijk}$ of a third-order tensor $𝓧\in {\mathbb{R}}^{I\times J\times K}$, can be seen as a state information matrix that stores the data collected from IoT sensors for object *i* at timestamp *j* and location *k*. The state information matrix should include the data collected and their types, which could be denoted by Unicode for instance. Two examples of element ${𝓧}_{ijk}$ are shown in Figure 5 for demonstration purpose, where object *i* is a man wearing a smart watch that automatically connects to the IoT network in smart communities. The health-related data collected, such as body temperature and heart rate, are then transmitted to the gateway in the IoT network and saved to the corresponding state information matrix. Object $i^{\prime }$ is a surveillance camera installed, and thus the data collected are video frames, each of which is usually represented by a third-order tensor. It is can be seen that the data in a state information matrix is not limited to a scalar, but also could be a matrix or even a tensor.

By forming such a third-order tensor to store the massive data collected from intelligent sensors in a smart community, the geometry structures of various types of data are preserved, which could be further exploited by tensor-based data processing methods to enhance data mining and analysis and thus assist smart community decision-making in many aspects, such as emergency management, human resource allocation, crime detection and forecasting, etc. For instance, based on the proposed tensor model, people can be identified via face recognition and tracked via object tracking techniques [23], based on which, the trajectory of a person can be obtained and analyzed. Once abnormal behavior is detected, the face image and trajectory information of the suspect will be sent back to the community management team via the IoT network for further investigation and/or possible face-to-face questioning. If a crime happened, the face image of the suspect would be then sent to the police station to help with identity and crime record checking. 

In the following, let us take a closer look at the mode-1 slices (time-location), mode-2 slices (object-location), and mode-3 slices (object-time) one by one.

**Figure 6:**
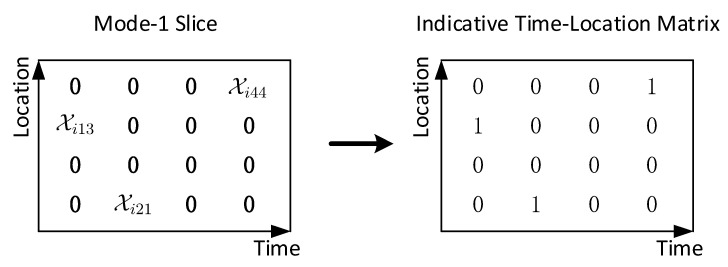
A toy example of mode-1 slice ${𝓧}_{i\;\;:\;\;:}$ and the corresponding indicative time-location matrix for object *i*.

- **Mode-1 (Time-Location) Slices **${𝓧}_{i\;\;:\;\;:}$: A time-location object slice ${𝓧}_{i\;\;:\;\;:}$ is a subarray of tensor $𝓧\in {\mathbb{R}}^{I\times J\times K}$ maintaining the time and location information for object *i*, where element ${𝓧}_{ijk}$, i.e., the state information matrix at timestamp *j* and location *k* for object *i* introduced above, indicates whether object *i* is within the *k*th subarea or not at timestamp *j*. Specifically, if ${𝓧}_{ijk}$ is a nonzero matrix, object *i* is located in the *k*th subarea; otherwise, it is outside subarea *k*. The location information can be obtained by using one or multiple localization technologies, e.g., Wi-Fi signal sensing, image recognition, etc. Note that a large object, for example a building, could appear in multiple subareas, resulting in more than one state information matrices in a location fiber. By retrieving a time-location slice from the tensor model, an indicative matrix for object *i* can be formed, which consists of 1 s and 0 s, as shown in Figure 6, where 1 and 0 denote that the corresponding state information matrix is a nonzero matrix or a zero matrix, respectively. This indicative time-location matrix would be very useful for trajectory analysis and object localization.
- **Mode-2 (Object-Location) Slices**$\;{𝓧}_{\;:\;j\;\;:}$: Similarly, an object-location slice ${𝓧}_{\;:\;j\;\;:}$ is a subarray that consists of object and location information collected at timestamp *j*. By detecting whether an element, i.e., the state information matrix of a mode-2 slice is a zero matrix or not, and using 1 to denote a nonzero matrix and 0 to denote a zero matrix, an indicative object-location matrix can be obtained, which clearly shows the geographical locations of various objects in the whole area at a given timestamp, which could be used to monitor social distancing state in a smart community/city in real time during the current COVID-19 epidemic.
- **Mode-3 (Object-Time) Slices **${𝓧}_{\;\;:\;\;:\;k}$: An object-time slice ${𝓧}_{\;\;:\;\;:\;k}$ is a subarray representing the state information of various objects at various timestamps at a given subarea. In other words, it shows what objects appear in a given area at what time and its state evolution with time. Such object-time slices are especially useful for resident-visitor classification. Specifically, a resident appears regularly at the entrance and exit, while a visitor usually appears randomly and might appear only a couple of times. Tracking of visitors can be then conducted in the cloud center in case of any crimes happened.

**Figure 7:**
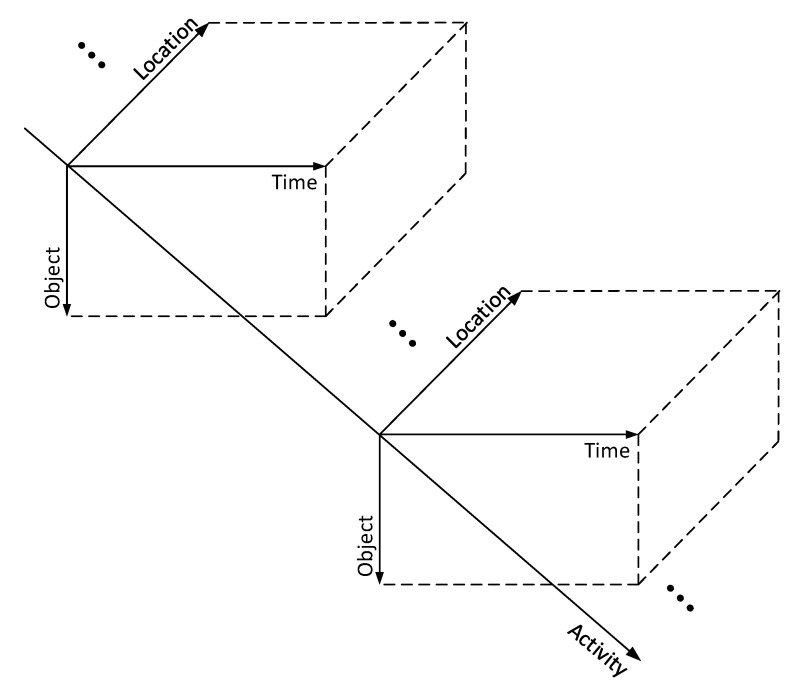
Graphic illustration of the proposed fourth-order tensor model for smart communities.

The above third-order tensor model can only show the objective components, i.e., object, time and location. However, the data stored in the tensor highly depend on human activity, as our society is naturally human-centered. Therefore, we propose to add a novel fourth dimension that represents human activity, which is referred to as activity in the following for simplicity. As such, a comprehensive fourth-order tensor model is established for smart communities, as depicted in Figure 7. Note that the proposed fourth-order tensor model could better describe real scenarios and thus help with more accurate decision-making and meeting more complex application requirements in smart communities. 

The activity dimension indices represent various human activities, which, in contrast to the other three dimensions, cannot be obtained directly from intelligent sensors. Instead, preprocessing the object-time-location tensor by using various machine learning algorithms is usually required. A number of common or dangerous human activities can be predefined, such as walking, running, gathering, falling, etc.

### 2.3. A Unified Fourth-Order Tensor-Based Smart Community System

**Figure 8:**
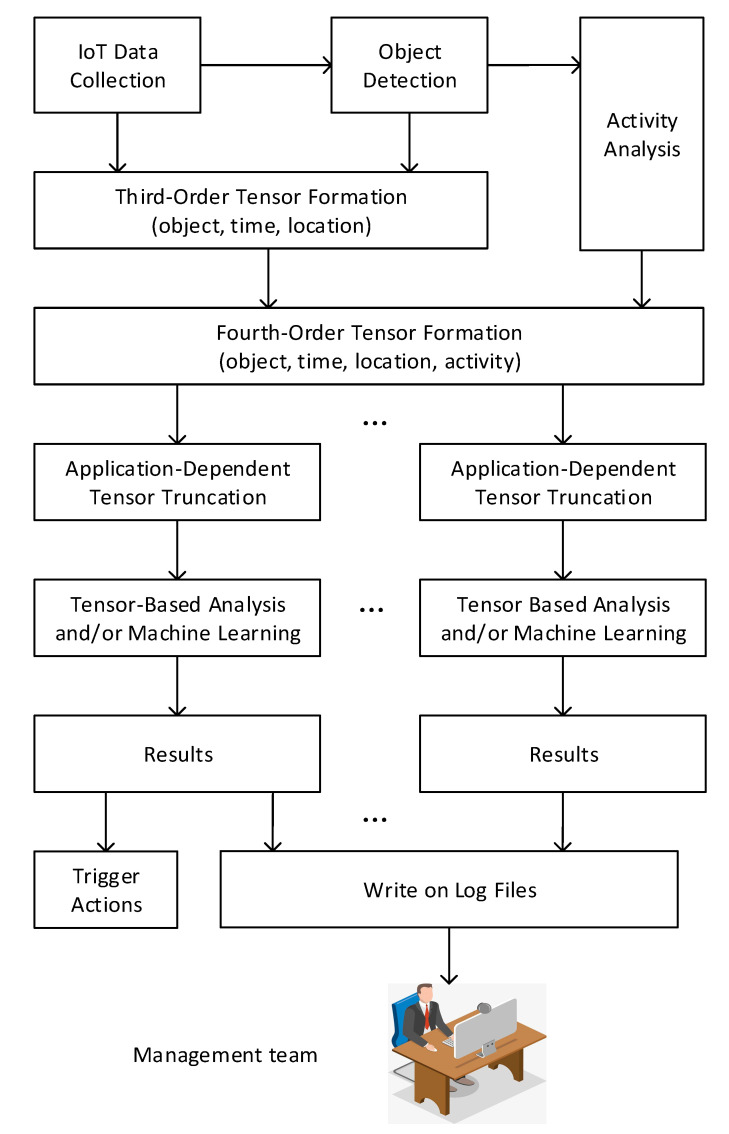
Block diagram of the proposed unified fourth-order tensor-based smart community system.

Based on the fourth-order tensor model that encodes object, time, location and human activity information, a unified smart community system is proposed in this section, as shown in Figure 8. Specifically, data collected from intelligent sensors, surveillance cameras and other devices are first sent to the gateways in the IoT network and then stored and processed in the cloud center. At the cloud side, real-time object detection is conducted based on the video frames fed back from surveillance cameras. By continuing to track an object once it is detected, the data collected for this object are formalized into a third-order tensor with 3 dimensions, being object, time and location, and then saved to the cloud storage. As this third-order tensor builds up, human activity analysis is being done simultaneously for classification. According to the human activity information, the third-order tensor data are reorganized into a fourth-order tensor so as to highlight human activity, which is useful for the applications that aim to investigate its effects and the relationship between human activity and other components.

So far, a fourth-order tensor has been constructed, from which useful data for specific applications with various purposes could be truncated over a certain range of objects, timestamps, locations and activities for further processing. These truncated tensor data can then be carefully analyzed to obtain insightful information or serve as the input of a machine learning framework with a specific purpose. Analytical and/or machine learning results will then be obtained and written onto the log files, which are monitored and reviewed by the management team to assist decision-making and improve the livability of the community. Some analytical and/or machine learning results could also trigger actions directly if required by the corresponding application, e.g., fire alarms will be triggered immediately if the obtained results suggest a suspicious fire. Since the fourth-order tensor stores all the sensed data, applications with new functions can be added to the existing smart community system. In other words, the proposed unified fourth-order tensor-based smart community system embraces flexibility and extendibility.

## 3. Application Examples

In this section, two exemplary applications will be introduced to demonstrate the flexibility of the proposed unified fourth-order tensor-based smart community system. 

### 3.1. Intelligent Traffic Light Control

Intelligent traffic light control at intersections, which adapts to road traffic including vehicles and pedestrians, can improve everyone’s experience. This is one of the benefits we could have in smart communities. To make it happen, accurate traffic forecasting is required, which can be obtained by using our proposed fourth-order tensor-based smart community system. 

Specifically, at an intersection, the traffic in a given period of time is related to that in other time periods around it, as people’s daily routine usually varies slightly over time. In order to control traffic light intelligently, the comprehensive information road traffic needs to be predicted, i.e., how many vehicles and pedestrians on which road will be turning right, going straight and turning left. In this case, the objects are the drivers and pedestrians; the timestamps are a number of time periods; the locations are the roads; and the activities are drivers’ and pedestrians’ actions, including turning left, turning right and going straight. Each element of the fourth-order tensor is either 1 or 0, which indicates whether an object at a specific timestamp and a specific location is taking a specific action. As there are so many elements in the fourth-order tensor space, each of which corresponds to one combination of object, timestamp, location and activity, the tensor is usually sparse, implying that the original tensor could be compressed to reduce the size and hence the amount of data so as to speed up further processing. A popular way is to find out a core tensor to represent the original one. 

By denoting the original tensor as $𝓧\in {\mathbb{R}}^{I_{1}\times I_{2}\times I_{3}\times I_{4}}$ and applying Tucker decomposition [24], tensor 𝓧 is decomposed into a core tensor $𝓖\in {\mathbb{R}}^{J_{1}\times J_{2}\times J_{3}\times J_{4}}$ and four factor matrices $U_{1}\in {\mathbb{R}}^{I_{1}\times J_{1}},\;U_{2}\in {\mathbb{R}}^{I_{2}\times J_{2}},\;U_{3}\in {\mathbb{R}}^{I_{3}\times J_{3}},U_{4}\in {\mathbb{R}}^{I_{4}\times J_{4}}$ with

(2) $$𝓨=𝓖\times_{1}U_{1}\times_{2}U_{2}\times_{3}U_{3}\times_{4}U_{4}.$$

A typical algorithm to calculate the core tensor and also the factor matrices is the higher-order singular value decomposition (HOSVD) algorithm [25]. After obtaining the historical core tensors of the truncated tensors over the same time period of interest in the past a few months, the historical core tensors can be used as training data to train a machine learning model to predict the core tensor ${𝓖}^{\prime }\in {\mathbb{R}}^{J_{1}\times J_{2}\times J_{3}\times J_{4}}$ of road traffic. Based on the core tensor ${𝓖}^{\prime }$, the predicted tensor ${𝓨}^{\prime }\in {\mathbb{R}}^{I_{1}\times I_{2}\times I_{3}\times I_{4}}$, which encodes the predicted road traffic information can be obtained. These predicted road traffic data can then be used for real-time traffic light control. 

In practice, four surveillance cameras need to be deployed in the intersection to monitor four crossroad areas. Real-time videos from these four cameras are sent back to the cloud center for object detection and activity analysis, based on which a fourth-order tensor could be formed. The above traffic forecasting algorithm can then be run in the cloud center to predict traffic in the coming minute, i.e., how many vehicles and pedestrians on which road are taking what actions. With the detailed road traffic information, an intelligent traffic control algorithm will be executed to control traffic light. 

### 3.2. Human Behavior Pattern Analysis

Thanks to the multi-dimensional data available in the tensor model, hidden behavior patterns of individuals can be extracted from the huge amount of data collected every day, which could provide statistical support for meeting people’s living needs. 

In order to analyze individual human behaviors, let us retrieve the third-order tensor ${𝓧}_{i\;:\;:\;:\;}\in {\mathbb{R}}^{I_{2}\times I_{3}\times I_{4}}$ for each human object *i* and construct a corresponding indicative matrix ${𝓜}_{i\;:\;:\;:\;}\in {\mathbb{R}}^{I_{2}\times I_{3}\times I_{4}}$, which includes time, location and activity information. By applying the canonical polyadic (CP) decomposition technique [26], which is another popular tensor decomposition method that approximates a tensor by the sum of a number of three-way outer products, we have

(3) $${𝓜}_{i\;:\;:\;:\;}\approx \overset{R}{\underset{r=1}{\sum }}a_{ir}\circ b_{ir}\circ c_{ir},$$

where *R* is the number of decomposed components, which can also be seen as the number of basic behavior patterns. For each basic behavior pattern *i*, $a_{ir}\in {\mathbb{R}}^{I_{2}}$, $b_{ir}\in {\mathbb{R}}^{I_{3}}$ and $c_{ir}\in {\mathbb{R}}^{I_{4}}$ indicate the distribution of pattern *i* in the dimensions of time, location and activity, respectively. It should be noted that the number of decomposed basic behavior patterns *R* is a key parameter that determines the ability of behavior pattern mining, which should be carefully chosen. 

Based on the distribution information obtained over time, location and activity, human behavior patterns can be analyzed from different points of view to fit different needs. For example, individuals’ behavior patterns regarding activity can be obtained, based on which individuals can be clustered into a number of groups of common interests, which could be utilized to meet people’s social needs. For the single inhabitants, a similarity check between one’s current behavior pattern and the past ones can be performed to detect abnormal events in case of any emergency needs. 

In practice, the time and location information of the residents in a smart community are collected from surveillance camera videos and smart home devices, based on which each resident’s activities are detected. The information is then organized into the fourth-order tensor model and stored in the cloud storage. When a public community event is planned, the truncated third-order tensor data for every resident could be analyzed by adopting the above human behavior pattern analysis method to identify interested residents, e.g., promoting interested residents to participate in weekly badminton training. For the purpose of abnormal event detection, the behavior pattern of a resident could be checked against those in the past days. Once a mismatch is detected, the community management team will message or ring the resident for a security check.

## 4. Discussion

In the last section, two exemplary applications have been presented to demonstrate the operation of our proposed fourth-order tensor-based unified smart community system. It can be seen that our unified smart community system can support various functionalities by retrieving interested data from the fourth-order tensor model of object, time, location and human activity and applying designated data processing methods. Such flexibility makes the smart community system easier to be upgraded with new functions. As a result, no removal of old intelligent systems and redeployment of new ones is needed, which reduces the maintenance cost significantly. In addition, since our proposed smart community system provides a unified platform for various applications with different goals, hardware devices can be shared by multiple applications, which is environmental-friendly in terms of both energy consumption and hardware manufacturing.

Moreover, as our proposed fourth-order tensor model includes more data, particularly human activity data, gains could be achieved from the proposed scheme compared to conventional methods based on third-order tensors. A representative third-order tensor-based scheme was proposed in [19] to forecast the rental data of urban public bicycles to assist redistribution. The three dimensions of the third-order tensor in [19] are time, location and numbers of bicycles rented out and returned back, while the effect of human activity is overlooked. It should be noted that human activity plays a key role in the usage of public bicycles. For example, a large number of university students might cycle together to a place for a big gathering. With such an accidental event, if human activity is not considered, the predicted number of public bicycles required around the gathering place based on previous data could be much smaller than the demand. As a result, a large number of students might not be able to cycle back to the campus after gathering. By adopting our fourth-order tensor model with one dimension representing human activity, student activity can also be predicted, which could be further exploited to optimize public bicycle redistribution to improve user experience.

Apart from the above advantages, the use of the tensor model to store all the collected data can preserve the geometry structures of multiple dimensions, indicating that additional hidden information could be exploited by adopting data mining techniques. Moreover, as the tensor model encodes multidimensional data of various types, in contrast to conventional methods which can usually handle single-parameter tasks, by adopting suitable tensor-based algorithms/methods a set of parameters can be predicated as a whole. Nevertheless, designing tensor-based algorithms to fit specific purposes could be very challenging and needs to be carefully investigated. Although the above benefits can be harvested thanks to the ability of a tensor to embed multi-dimensional data, it also brings challenges. For example, a high-order tensor usually has enormous elements, which require high computing capability to process data. Efficient tensor decomposition algorithms have been recently proposed in [27,28,29], which can achieve up to 14 times faster tensor decomposition. How to further accelerate tensor decomposition and reduce tensor data size as far as possible is still an open problem, which deserves significant efforts in future work. 

## 5. Conclusions

In this paper, a novel fourth-order tensor model was proposed to formalize the data collected from intelligent sensors in smart communities, based on which a unified smart community system was proposed. By presenting two exemplary applications, it was shown that by truncating the tensor data according to application requirements, various applications with different functionalities can be integrated into the same smart community system. More importantly, new applications with new functionalities can be easily added to the existing system if needed, indicating that our proposed fourth-order tensor-based smart community system is flexible and extendable. 

Note that this paper mainly discussed the fourth-order tensor-based theoretical framework to enable a unified smart community system. In the future, the effectiveness and efficiency of our proposed system will be carefully investigated and compared against conventional methods by implementing a number of functions in practice.
